# Recent advancements in pediatric purulent meningitis: diagnosis and treatment

**DOI:** 10.1186/s13052-025-02192-4

**Published:** 2026-01-07

**Authors:** Lingrong Yang, Wenwen Jin, Yanli Liu, Wei Hu, Yu Chen, Guoyi Wang, Wen Li, Qiqi Gao

**Affiliations:** 1 Sichuan Provincial Maternity and Child Health Care Hospital, Sichuan Provincial Women’s and Children’s Hospital, The Affiliated Women’s and Children’s Hospital of Chengdu Medical College, Sichuan, Chengdu, 610045 China; 2 The Second Affiliated Hospital, Yuying Children’s Hospital of Wenzhou Medical University, Wenzhou Medical University, Wenzhou, Zhejiang 325000 China; 3 Minerva Hospital for Women and Children, Chengdu, Sichuan 610045 China

**Keywords:** Children, Purulent meningitis, Pathogenesis, Early recognition, Treatment

## Abstract

Purulent meningitis is a common disease of the central nervous system affecting children. It arises from various pathogens transmitted through diverse routes and can lead to significant sequelae, even death. The pathogenesis involves varying degrees of meningeal damage by pathogens, breaching the blood-brain barrier (BBB) and triggering immune responses and other physiological processes. Early diagnosis of purulent meningitis relies on recognizing clinical symptoms and conducting comprehensive examinations in patients. This includes young children presenting with systemic symptoms of infectious toxemia, neurological abnormalities, C-reactive protein (CRP), and procalcitonin (PCT) levels. Additionally, examination of cerebrospinal fluid (CSF) for abnormalities, identification of bacterial pathogens at the genetic level, brain computed tomography (CT) and magnetic resonance imaging (MRI) findings are crucial. Culturing bacteria from CSF remains the gold standard for diagnosing purulent meningitis. Current treatment approaches primarily involve antibacterial drugs, supportive therapy, and immunotherapy. Promising advancements are being made in exploring further treatment options and strategies. It is noteworthy that poor prognosis in pediatric patients can be inferred from clinical manifestations, laboratory findings, and imaging results. This review expounds on the recent progress in understanding the pathogenesis, early recognition, treatment strategies, and risk factors associated with poor outcomes in purulent meningitis affecting children (See Graphical Abstract).

**Graphical Abstract:**

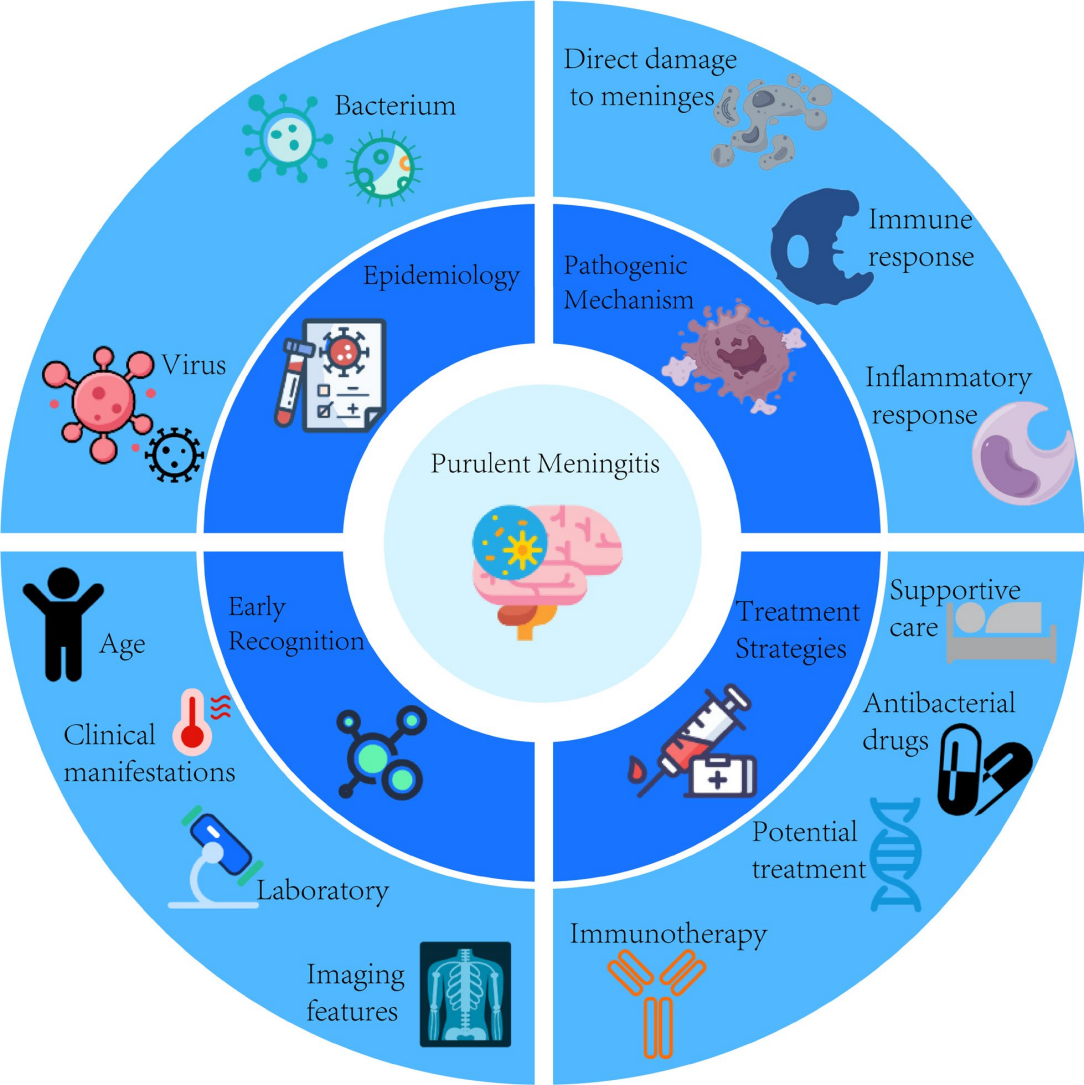

## Introduction

Purulent meningitis is an infectious disease caused by various bacteria that invade the meninges, the membranes surrounding the brain and spinal cord. It can affect individuals of all ages, with a relatively high incidence as documented in several studies [1, 2]. The Global Burden of Disease (GBD) 2021 Collaboration on Neurological Diseases states that the incidence of meningitis in 2021 is approximately 92.3 per 100,000 people [3]. Newborns are of particular concern as the morbidity and mortality due to purulent meningitis in this population remain high [4].

Despite advancements in medical technology and incremental progress in purulent meningitis research, significant challenges remain in the diagnosis and treatment of this patient population. The disease exhibits a high incidence in children, especially newborns, with premature infants facing a particularly high risk. Studies report an incidence of up to 3 per 1,000 in premature infants compared to 0.12–1 per 1,000 in full-term infants [5–7]. Without timely and effective treatment, children with purulent meningitis may develop a range of sequelae, including hydrocephalus, epilepsy, hearing loss, motor dysfunction, visual impairment, behavioral and emotional disorders, and intracranial structural damage. These complications can even lead to death [8, 9]. According to the GBD 2019 Meningitis Antimicrobial Resistance Collaborators analysis, the mortality rate is 16.9 per 100,000 deaths among under-5 meningitis and 137.2 per 100,000 deaths among neonatal meningitis [10]. Therefore, a comprehensive understanding of diagnosis and treatment advancements in childhood purulent meningitis is crucial for improving children’s quality of life and prognosis. This article focuses on the pathogenesis, early identification, treatment options, and risk factors associated with poor outcomes in this disease. We aim to raise awareness among clinicians, encourage further research, and ultimately contribute to enriching treatment plans, reducing the occurrence of sequelae, and mitigating their severity in children.

## Epidemiology

Purulent meningitis can be caused by a variety of pathogenic bacteria, including *Streptococcus agalactiae* (Group B *Streptococcus*, GBS), *Escherichia coli (E. coli)*,* Staphylococcus aureus (S. aureus)*,* coagulase-negative staphylococcus*,* Neisseria meningitidis (N. meningitidis)*,* Streptococcus pneumoniae (S. pneumoniae)*,* Haemophilus influenzae (H. influenzae)*,* Klebsiella pneumoniae (K. pneumoniae)*,* Listeria monocytogenes (L. monocytogenes)*, and *Kurthia*, among others [4, 11–16]. Specific serotypes of these pathogens demonstrate a robust association with purulent meningitis, highlighting their critical role in the condition. From a clinical perspective, the primary route of infection in purulent meningitis involves hematogenous dissemination. These bacteria can originate from either the external environment or sites of colonization [17–19]. Here, we focus on several key types of pathogenic bacteria (see Table 1 in the End of Document):

**Table 1:** Characteristics of different types of pathogens

Pathogen	Highly correlated serotypes	Virulence factors	Clinical manifestations	Vulnerable age groups	Reference
*Streptococcu Pneumoniae*	19 F, 14, 23 F, 6 A, 6B, 19 A	Capsular polysaccharides, Surface proteins, Pneumolysin, Neuraminidase, etc.	Spread primarily by droplets and can colonize the nasopharynx in normal people.	Children under 2 years of age	[20–24]
*Haemophilus Influenzae*	B	IgA1 protease, Outer membrane vesicles (OMVs), Capsule polysaccharide, Pili, Macrophage survival factor, etc.	Spread mainly by droplets, the mortality was significantly high before vaccine introduction.	Children under 5 years of age	[25–30]
Group B *Streptococcus*	Ⅲ-4, ST17	Capsule, β-hemolysin/cytolysin, C5a peptidase, Adhesins, Two-component systems, Membrane vesicles, etc.	Spread by maternal infection or genital infection.	Infants under 3 months of age	[31–35]
*Neisseria Meningitidis*	B, C, W, X, Y	Capsule, Outer membrane vesicles (OMVs), Factor H binding protein, Pili, Opacity-associated adhesion proteins, Surfactant proteins, etc.	Spread by droplets and can rapidly progress to life-threatening conditions.	Incidence peaks in infants and adolescents; age distribution varies by serogroup and during pidemics.	[27, 36–46]
*Listeria Monocytogenes*	4B	Internalins, Listeriolysin O, Phospholipases, Actin assembly-inducing protein, etc.	Spread by fecal-oral transmission or contact transmission.	Neonates	[47–50]

*S.pneumoniae*, a Gram-positive bacterium that colonizes the nasopharynx, is the leading cause of purulent meningitis in children, particularly those under 2 years of age. It is spread primarily by droplets and can also cause purulent meningitis in severe cases [20, 21]. It has many serotypes that can cause disease, including 19F, 14, 23F, 6A, 6B, 19A and many others [20, 22]. The virulence factors of *S. pneumoniae* include capsular polysaccharides, surface proteins (A and C), pneumolysin, neuraminidase, and complement evasion mechanisms [23]. With the popularization of pneumococcal conjugate vaccine, the proportion of non-vaccine serotypes increased gradually [24].

*H. influenzae*, a Gram-negative bacterium, is classified into six serotypes (A to F), with *Haemophilus influenzae* serotype b (Hib) representing a major pathogenic strain [25]. Hib is a significant causative agent of purulent meningitis, a severe condition particularly prevalent among children under five years of age [26]. The virulence factors of *H. influenzae* include IgA1 protease, outer membrane vesicles (OMVs), capsule polysaccharide, pili, and macrophage survival factor [27–29]. Transmission predominantly occurs via respiratory droplets, with an estimated incidence rate of 1.13 cases per 100,000 individuals. Prior to the widespread implementation of the Hib vaccine, the mortality rate associated with Hib infections was notably high [30].

*Group B Streptococcus* (GBS), also known as *Streptococcus agalactiae*, is a significant pathogen associated with neonatal intracranial infections, particularly in infants under three months of age [31–33]. Transmission can occur from mother to newborn either before birth or during delivery through the birth canal. GBS comprises ten serotypes: Ia, Ib, II, III, IV, V, VI, VII, VIII, and IX. Notably, serotypes Ia, Ib, and III are frequently identified in southern mainland China, where the hypervirulent Sequence Type 17 (ST17) is widely distributed [33, 34]. The virulence factors of GBS include the capsule, β-hemolysin/cytolysin, C5a peptidase, adhesins, two-component systems, and membrane vesicles [35].

*N. meningitidis*, a nasopharyngeal commensal, is another significant pathogen responsible for epidemic meningitis, with incidence peaking in infants and adolescents, with the distribution varying by serogroup and during epidemics [36]. It is primarily transmitted through respiratory droplets and can rapidly progress to life-threatening conditions, with mortality potentially occurring within hours of symptom onset. Even with appropriate treatment, the case-fatality rate remains substantial, ranging from 8% to 15% [37, 38]. This bacterium can be categorized into twelve serogroups, with serogroups B, C, W, X, and Y demonstrably linked to purulent meningitis [39–41]. The virulence factors of *N. meningitidis* include the capsule, OMVs, factor H binding protein, pili, opacity-associated adhesion proteins, and surfactant proteins [27, 42]. The prevalence of these serogroups varies geographically. For instance, Chile predominantly experiences meningitis caused by serogroup W, while Niger faces a higher burden from serogroup C. In China, serogroups C, B, and W are the most prevalent [39, 43, 44]. Infections caused by *N. meningitidis* are particularly prevalent in Africa, where they have been associated with multiple outbreaks. Prior to the introduction of the meningococcal serogroup A conjugate vaccine, approximately 90% of meningitis cases in the region were attributed to *N. meningitidis* serogroup A [45, 46].

*L. monocytogenes*, a foodborne Gram-positive bacterium, poses a risk beyond gastrointestinal illness, as it is capable of causing central nervous system infections [47]. The primary route of transmission is through the consumption of contaminated food; however, infections have also been reported to occur via direct contact [48]. Furthermore, pregnant women infected with *L. monocytogenes* can transmit the pathogen to their newborns, leading to neonatal infections [49]. This pathogen accounts for approximately 4–5% of purulent meningitis cases in infants under 90 days old and 1.5% in neonates within the first 28 days of life. Notably, among the 13 identified serotypes of *L. monocytogenes*, serotype 4b is particularly associated with purulent meningitis cases. The virulence factors of *L. monocytogenes* include internalins, listeriolysin O, phospholipases, and actin assembly-inducing protein [50].

The emergence of the novel coronavirus, severe acute respiratory syndrome coronavirus 2 (SARS-CoV-2), has profoundly impacted global health in recent years. A subset of pediatric patients infected with SARS-CoV-2 may develop purulent meningitis as a complication of secondary bacterial infections [51].Notably, during the COVID-19 pandemic, studies reported a reduced incidence of purulent meningitis among febrile infants testing positive for SARS-CoV-2, a phenomenon potentially attributable to the widespread implementation of preventive measures during the outbreak [52–54].

It is now understood that purulent meningitis exhibits no clear seasonal pattern, with cases occurring throughout the year [55]. The geographic distribution of the disease also remains under investigation, although some regional variations in pathogen prevalence have been identified. For instance, *L. monocytogenes* appears to be more prevalent in African countries [56, 57]. The complex and multifaceted epidemiology of purulent meningitis, involving diverse pathogens and influencing factors, necessitates a comprehensive understanding to guide effective prevention and treatment strategies.

## Pathogenesis

### Pathogenic bacteria cause direct damage to the meninges

The blood-brain barrier (BBB) serves as a critical natural defense, shielding the brain from pathogens by separating plasma from cerebrospinal fluid (CSF). To inflict damage on the meninges, these pathogens must first breach this barrier, a process that relies on specific interactions between microbial and host factors or signaling molecules [58–60]. *E. coli*, for instance, can exploit two pathways to invade the BBB: the epidermal growth factor and cysteinyl-leukotriene pathways. Additionally, it can disrupt the BBB through its α-hemolysin toxin, which diminishes non-classical hedgehog (HH) signaling in brain microvascular endothelial cells by reducing transforming growth factor beta receptor II (TGFBRII)/glioma-associated oncogene homolog 2 (Gli2) activity [61, 62]. Furthermore, *E. coli* can trigger the upregulation of the host transcription factor early growth response 1, leading to the activation of Ras homolog gene family member A, Ras-related C3 botulinum toxin substrate 1, and Cell Division Control protein 42. This cascade ultimately induces the expression of vascular endothelial growth factor A, platelet-derived growth factor subunit B, and angiopoietin-like 4. These factors induce cytoskeletal changes and the degradation of tight junction proteins, ultimately compromising the integrity of the BBB [63]. *E. coli* K1, in particular, utilizes interactions with host receptor proteins to facilitate its penetration of the BBB and stimulate the release of endogenous ligands, factors closely associated with its ability to traverse the barrier [64, 65]. *S. pneumoniae* and GBS are widely thought to employ a distinct mechanism for BBB invasion, potentially involving transferrin. These pathogens may exploit the fusion of bacteria-containing vesicles with transferrin vesicles to cross the BBB through a process known as entosis [66]. Similarly, in *Klebsiella pneumoniae*, Research shows that its virulence factor OmpA promotes BBB penetration via a transcellular pathway, a “Trojan horse” pathway using macrophages, and a pro-inflammatory pathway, demonstrating a highly adaptable and potent invasion strategy [67].

Following the successful breach of the BBB, pathogens can trigger brain arteriovenous lesions and inflammation. Upon reaching the child’s brain, they activate a multitude of immune recognition cells. Microglia, a type of immune cell, transform into the M1 phenotype after activation, leading to the production and release of pro-inflammatory cytokines like Tumor necrosis factor-α(TNF-α), Interleukin-1β, and Interleukin-6. These inflammatory cytokines trigger a detrimental cascade effect. They not only disrupt the BBB, increasing cerebral vascular permeability and causing vasogenic cerebral edema but also exacerbate the inflammatory response within brain tissue. This can lead to irreversible damage such as cellular cerebral edema, blockage of small blood vessels, and nerve cell necrosis. Additionally, inflammatory cytokines promote the accumulation of polymorphonuclear neutrophils in the brain to clear pathogens. However, this influx can further contribute to neuronal atrophy and brain damage [68]. Beyond this immunopathology, certain pathogens like *Streptococcus pneumoniae* directly damage neurons. The pore-forming toxin pneumolysin (Ply) induces direct cellular lysis and, together with the RrgA adhesin, binds neuronal β-actin, leading to invasion, cytoskeletal disassembly, and neuronal death [69, 70]. Notably, oxidative stress also plays a role in neuronal injury. Activation of signaling pathways like mitogen-activated protein kinase can trigger apoptosis and free radical production, ultimately worsening disease progression [71].

### Pathogenic bacteria can activate the body’s immune function

Toll-like receptors (TLRs) are pivotal in initiating the innate immune response to CNS pathogens by recognizing pathogen-associated molecular patterns, a process which, in pediatric meningitis, involves a global upregulation of TLR genes and their downstream signaling molecules in the cerebrospinal fluid, ultimately driving a potent inflammatory response [72]. However, pathogens also possess strategies to evade or suppress the immune response. In patients with purulent meningitis, early CD4^+^CD25^+^ regulatory T cells - a type of immune cell that dampens inflammation - may be suppressed, potentially leading to a more severe inflammatory response. The exact mechanism behind this remains unclear [73]. *E. coli* employs another strategy to evade the immune response. It releases transforming growth factor β1 from outside the cell, which upregulates intracellular miR-155. This microRNA, in turn, activates non-classical HH signaling, ultimately suppressing the immune response of endothelial cells to infection [74]. Interestingly, *E. coli* K1 utilizes its lipopolysaccharide to inhibit the activation of nicotinamide adenine dinucleotide phosphate (NADPH) oxidase in neutrophils, thereby reducing the production of reactive oxygen species - potent antimicrobial molecules [68, 75]. *S. pneumoniae* leverages its polysaccharide capsule, a key virulence factor, to achieve immune escape. This capsule can manipulate the autophagy recognition mechanism, another strategy for the bacterium to evade the host’s immune response [76].

Beyond localized effects, pathogen invasion can trigger complex physiological processes throughout the body, including hormonal disruptions and systemic inflammatory response syndrome (SIRS) [77]. SIRS is believed to be a significant contributor to patient morbidity (injury) in purulent meningitis. However, its specific role in this disease requires further investigation.

Purulent meningitis arises from a complex interplay of factors and biological processes. These include breaching the BBB, triggering an inflammatory response, immune cell activity, and hormonal disruptions. Indeed, a comprehensive understanding of the interactions and regulatory mechanisms between these elements is crucial for developing effective preventive and therapeutic strategies (see Table 2 in the End of Document).

**Table 2:** Mechanisms of purulent meningitis

Pathogen entry		Immune reaction
Through the blood-brain barrier(BBB)	After the process	
α-hemolysin attenuating transforming growth factor beta receptor II (TGFBRII)/glioma-associated oncogene homolog 2 (Gli2)-mediated signaling [62]	Activate immune recognition cells (small keratinocytes, etc.) and release proinflammatory cytokine [68]	Toll-like receptor(TLR) recognition induces an innate immune response [72]
the activation of early growth response 1 leads to induced cytoskeletal changes and downregulated tight junction protein expression [63]	Polymorphonuclear neutrophil (PMN) concentrated in the brain [68]	CD4 + CD25 + regulatory T cell are suppressed [73]
epidermal growth factor receptor and cysteinyl leukotrienes for invasion [61]	Mitogen-activated protein kinase and other signaling pathways are activated [71]	exogenous Transforming growth factor β1(TGF β1) and non-classical Hedgehog (HH) signals to suppress immunity [74]
hitchhike on transferrin receptor (TfR) transcytosis to cross the BBB [66]	RrgA- and pneumolysin (Ply)-mediated binding to neuronal β-actin, leading to neuronal invasion and death [69].	lipopolysaccharide to reduce reactive oxygen species [68, 75]
OmpA-mediated BBB penetration via transcellular traversal, “Trojan horse” migration, and pro-inflammatory disruption [67].	Pore-forming toxin pneumolysin induces direct cellular lysis [70]	an autophagy recognition mechanism to escape [76]

## Early recognition

Early identification of purulent meningitis in children is paramount for timely treatment and improved prognosis. Given the variations in immune function and susceptibility to pyogenic bacteria across different age groups, children exhibit distinct clinical presentations. Therefore, incorporating age-specific considerations into the diagnostic process is critical to enhance early identification accuracy. Currently, the diagnosis of purulent meningitis in children primarily relies on a combination of factors: epidemiological history, clinical manifestations, laboratory examinations, imaging findings, and genetic detection methods. Here, we summarize various methods for the early identification of purulent meningitis in children.

### Age

It is well-established that variations in immune function across different age groups influence a child’s response to pyogenic bacteria. Furthermore, susceptibility to specific pyogenic bacteria also varies with age, with a general trend of decreasing prevalence as children grow older [31]. Infants younger than 3 months are particularly susceptible to *E. coli* and GBS infections, while children between 3 months and 5 years old are more prone to *S. pneumoniae* meningitis [31, 78]. *N. meningitidis*, another causative agent, exhibits the highest prevalence of intracranial infections in infants and young children [37]. Clinical presentations of purulent meningitis also differ by age group. Younger patients often exhibit non-specific symptoms like fever, jaundice, and poor feeding. They are also more susceptible to intracranial complications such as subdural effusion, brain abscess, and cerebral hemorrhage. In contrast, older children are more likely to present with typical signs of systemic symptoms of infectious toxemia, along with neurological manifestations such as headaches, vomiting, and altered consciousness [79, 80].

### Clinical manifestations

Purulent meningitis typically manifests with systemic symptoms of infectious toxemia, along with neurological abnormalities. Patients may experience some symptoms in the early stages, including fever, nausea or vomiting, and coughing, while in the late stages, the symptoms are characterized by central nervous system involvement, which may include neck stiffness, altered mental status (including blurred consciousness, irritability, drowsiness, and even coma), seizures, and headaches [81, 82]. The specific pathogen can influence the severity of symptoms, with some leading to complications like respiratory depression and distress [83]. Notably, neonates often present with more subtle clinical manifestations. They may not exhibit fever, classic signs of meningeal irritation (like neck stiffness), or a bulging anterior fontanelle, but instead show irritability and inability to be consoled [84]. Therefore, during diagnosis, physicians must carefully consider the mother’s pregnancy and birth history, including factors like amniotic fluid contamination, premature rupture of membranes, chorioamnionitis, and whether the baby is small for gestational age. These details can aid in the more accurate identification of potential risks [79, 85].

Fever, particularly acute fever, is a crucial indicator for early identification of purulent meningitis in children, especially newborns. Specific criteria and scoring models can be employed for preliminary screening and early detection. Among them, the Rochester Criteria (RC) and Yale Observation Scale (YOS) are well-established reference tools, while Lab-score and the Step-by-Step approach (both original and modified) have also demonstrated efficacy [86, 87]. The Boston strategy is regarded as the preferred option due to its cost-effectiveness [88]. Recently, the Pediatric Emergency Care Applied Research Network (PECARN) developed a new clinical prediction rule that identifies low-risk febrile infants aged 60 days and below with severe bacterial infection by urinalysis, absolute neutrophil count, and procalcitonin (PCT) levels. However, caution is needed when applying this rule to infants with durations of fever [89, 90]. Additionally, the Febrile Infant Triage Risk Score+ (FIRST+) has emerged as another effective evaluation tool, exhibiting high sensitivity and specificity by comprehensively considering the child’s age, body temperature, gender, duration of fever, urinary leukocyte esterase levels, and PCT levels [91]. It is worth noting that different tools perform variably across different age groups of patients. Therefore, during the diagnostic process, it is necessary to select appropriate assessment tools [92].

Fever is not the only warning sign in children. A rash on the body’s surface, especially in the early stages of the disease, also warrants close attention. Initially, there may be only one or two subtle petechiae that require careful examination, including the hands, feet, and conjunctiva for detection [79]. Studies have shown that the co-occurrence of fever and petechiae may be indicative of purulent meningitis [93]. In patients with petechiae, prompt evaluation to rule out meningitis is crucial to prevent serious complications like fulminant purpura and Waterhouse-Friderichsen syndrome [94]. In summary, the clinical presentations of purulent meningitis in children vary considerably. During diagnosis, physicians must consider a child’s age, medical history, clinical manifestations, and other relevant factors. A comprehensive judgment that integrates specific assessment tools and laboratory test results is essential for accurate diagnosis.

### Laboratory tests

Laboratory tests play a vital role in the early identification and diagnosis of purulent meningitis. Common laboratory examinations include complete blood count, erythrocyte sedimentation rate, and C-reactive protein (CRP) levels. These tests typically reveal significant elevations in white blood cells, CRP, and PCT. However, it is important to note that some pathogens can cause a decrease in white blood cell count (WBC). Therefore, interpreting laboratory results must be done in conjunction with clinical manifestations [83, 95–97]. Disseminated intravascular coagulation, a condition where blood clots abnormally throughout the body, can also indicate the presence of purulent meningitis [98]. CSF examination remains a cornerstone of diagnosing purulent meningitis. When a patient is suspected of meningitis, lumbar puncture should be performed immediately after clinical examination to obtain CSF [1, 99]. Analysis of CSF parameters, including WBC, neutrophil count, and the percentage of neutrophils, is crucial for diagnosis [100]. It is noteworthy that not all patients with meningitis will exhibit abnormal WBC in their CSF, but this situation is extremely rare [101]. Recent research has identified promising new diagnostic clues in CSF. These include changes in ferritin levels, fatty acid composition, matrix metalloproteinase 2 and its inhibitor 1, as well as decreased β2-microglobulin levels [97, 102–104]. For diagnosing neonatal purulent meningitis, TNF-α and heparin-binding protein (HBP) show significant value, with HBP demonstrating higher accuracy than traditional markers [105, 106]. As research continues to advance, new biomarkers and detection techniques are constantly emerging, offering improved possibilities for early identification of purulent meningitis. For instance, the prediction model for early diagnosis developed by Wu et al., which is based on PCT, CSF protein, and CSF glucose levels, provides a novel approach [107]. Complementing this, Sun et al. developed a model specifically for preterm infants, integrating birth weight, PCT within the first 24 h of life, cesarean section, and premature rupture of membranes to predict the risk of neonatal purulent meningitis [108]. Furthermore, the detection and application of neutrophil extracellular traps (NETs) in purulent meningitis has become a research hotspot in recent years, offering a new perspective for evaluating central nervous system infections [109–111].

CSF examination plays a pivotal role in the diagnosis of bacterial meningitis, with the culture of pathogenic bacteria representing a critical step. In addition to traditional culture methods, techniques such as Polymerase Chain Reaction (PCR), latex agglutination testing, and pathogen antigen identification are also utilized for the early detection of pathogenic microorganisms [112–114]. However, the latex agglutination test exhibits limited practicality in clinical settings due to its susceptibility to various factors, low diagnostic yield, and high cost [115, 116]. Advancements in gene detection technology have ushered in a new era of diagnostic tools. These technologies allow results to be available in a matter of hours. Recent studies in the United States have validated the efficacy of PCR-based methods in identifying specific pathogens within approximately three hours, exhibiting a sensitivity of 89.5% and a specificity of 97.4% for bacterial isolates [1]. Subsequently, the incorporation of mutant Taq DNA polymerase can significantly enhance the sensitivity of quantitative PCR techniques for targeting pathogens such as *S. pneumoniae*, *N. meningitidis*, and *H. influenzae* [117]. Genetic sequencing techniques, too, have emerged as a powerful diagnostic tool. High-throughput 16S amplicon sequencing has gained considerable momentum, owing to its improved sensitivity and specificity [118]. Meanwhile, next-generation metagenomic sequencing (mNGS) has demonstrated significant sensitivity and specificity in neonatal populations, offering valuable insights for clinical diagnosis [119–121]. Although mNGS may yield false-positive results and is associated with higher detection costs and longer processing times, its diagnostic utility remains substantial and should not be overlooked [122]. Notably, Zhao, Chengna et al. have developed a real-time PCR-based purulent meningitis-TaqMan array card. This platform can identify 21 pathogens, including *S. pneumoniae*, *S. aureus*, *E. coli*, GBS, and *N. meningitidis*, within three hours, boasting a sensitivity and specificity of 95% and 96%, respectively. This rapid and cost-effective alternative to mNGS offers significant advantages in clinical settings [123]. Concurrently, advancements in DNA microarray technology and the Bacterial Meningitis Scan continue to enhance their diagnostic capabilities, promising further refinement in the near future [124, 125]. In conclusion, laboratory examinations play a crucial role in the early identification and diagnosis of purulent meningitis. The continuous emergence and application of novel technologies and methods offer promising prospects for achieving more accurate and efficient diagnosis and treatment in the future.

### Imaging features

Imaging examinations play a certain role in the diagnosis and treatment of purulent meningitis. Magnetic resonance imaging (MRI) and computed tomography (CT) are two of the most commonly used methods. Although doctors tend to use CT initially, it may not be able to identify early changes. However, MRI can better distinguish soft tissues, which is conducive to observing early changes [126]. Kralik, S F, et al. believe that brain MRI has high sensitivity and moderate specificity in diagnosing bacterial meningitis [127]. Combining MRI findings with CSF biomarkers like TNF-α, lactate dehydrogenase, aspartate aminotransferase, and total protein can further enhance the accuracy of early diagnosis [105, 127]. In addition, imaging studies are also of great significance in identifying some complications of purulent meningitis or structural defects in the patient’s brain [128, 129]. Interestingly, imaging characteristics of purulent meningitis can vary depending on the causative pathogen. GBS infections are more likely to cause cerebral infarction, while *E. coli* infections are more commonly associated with hydrocephalus [130]. These distinctions offer valuable clues to clinicians, aiding in more precise pathogen identification and guiding treatment plans.

Advancements in image processing technology have led to the development of MRI methods based on immune clustering algorithms that demonstrate superior performance in diagnosing purulent meningitis compared to traditional techniques [131]. In addition, studies have revealed that children with purulent meningitis often exhibit abnormal electroencephalogram (EEG) findings, such as a significant decrease in the alpha/theta and alpha/delta rhythm power ratio. This finding offers a promising avenue for aiding in the diagnosis of the disease [132].

## Therapy

**Fig. 1:**
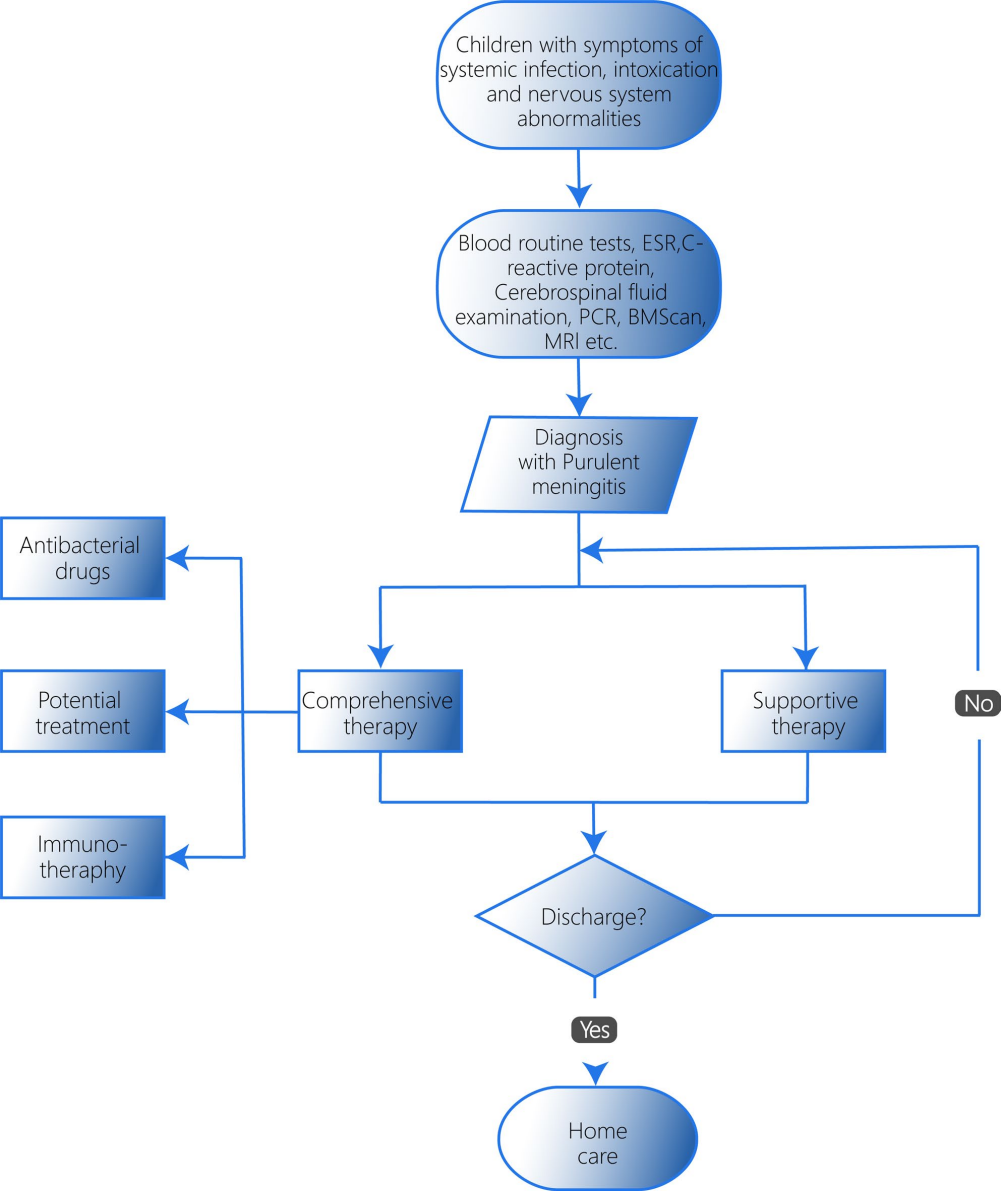
The process of therapy for purulent meningitis. The diagnosis of purulent meningitis is mainly based on the clinical manifestations and laboratory results of the child, in which fever is the most significant clinical manifestations. Treatment: (**a**) early empirical selection of bactericides that are easy to enter the cerebrospinal fluid; (**b**) Then adjust according to the drug sensitivity test results, clinical manifestations and blood and cerebrospinal fluid culture results; (**c**) The use of glucocorticoid pulse therapy; (**d**) prophylactic vaccination

The treatment of purulent meningitis is a multifaceted process. Clinicians must develop a personalized treatment plan for each patient, taking into account their specific condition, clinical manifestations, laboratory results, and imaging data. Here, we discuss the common treatment approaches employed in clinical practice (Fig. 1).

### Antibacterial drugs

Antibacterial therapy is paramount in treatment decisions for purulent meningitis. Due to the varied bacterial species involved, the targeted selection of antibiotics is critical. Commonly used antimicrobial agents include penicillins, cephalosporins, tetracyclines, macrolides, and others; however, their efficacy varies depending on the specific causative pathogen. There is a certain distinction in the susceptibility of pathogens among children of different age groups. Studies have shown that infants younger than 3 months of age are more likely to be infected with *E. coli* and GBS, whereas those over 3 months are more susceptible to *S. pneumoniae* [31, 78, 107]. When purulent meningitis is suspected, empiric broad-spectrum antibiotic therapy is typically initiated immediately, to prevent disease progression. According to both the 2017 Infectious Diseases Society of America (IDSA) guidelines for healthcare-associated ventriculitis and meningitis and the Chinese Expert Consensus on the Diagnosis and Treatment of Neurosurgical Central Nervous System Infections (2021 edition), vancomycin combined with an anti-pseudomonal β-lactam antibiotic (e.g., cefepime, ceftazidime, or meropenem) is recommended as the initial empirical treatment. Following the receipt of bacterial culture and susceptibility test results, antibiotic therapy should be adjusted to sensitive ones [133, 134]. Critically, when selecting antibiotics, preference should be given to those with good BBB penetration to ensure adequate CSF exposure. The duration of treatment varies depending on the specific pathogen, with potentially extended courses for neonates.

The extensive application of antimicrobial drugs has inadvertently resulted in the selection of bacterial strains harboring genetic mutations that confer resistance to these very drugs. Currently, certain *Escherichia coli* strains produce extended-spectrum β-lactamases (ESBLs), which inactivate a wide range of β-lactam antibiotics, resulting in resistance to penicillins, third-generation cephalosporins, and monocyclic β-lactams. A study by Chen, Yin-Ting et al. identified plasmid-mediated ESBLs in up to 9.1% of cases, underscoring the significance of this issue [96, 135–137]. This phenomenon underscores the critical need for continuous surveillance of bacterial resistance patterns to optimize therapeutic strategies. Ongoing research is being conducted to assess the drug resistance of different bacteria. For instance, a study by Nureen et al. investigating meningitis-causing pathogens in Pakistani children demonstrated that *E. coli* strains remained susceptible to imipenem and amikacin [138]. *S. pneumoniae*, previously sensitive to antibiotics such as penicillin and amoxicillin, has developed resistance in multiple strains [139]. Notably, *S. pneumoniae* in Brazil, Botswana, and Lima, Peru, have demonstrated significant resistance to penicillin, with high levels of resistance to ceftriaxone observed in all regions except Botswana [140–142]. In China, most children with pneumococcal purulent meningitis show sensitivity to vancomycin, linezolid, moxifloxacin, meropenem, rifampin, chloramphenicol, and ofloxacin but decreased sensitivity to penicillin and erythromycin [80, 143]. It is important to note that antibiotic use can be associated with adverse effects. For example, the most common and clinically significant side effect of vancomycin is renal dysfunction, and it may also lead to severe thrombocytopenia, neutropenia, and impaired liver function [144–147]. In light of these concerns, researchers are actively exploring novel antimicrobial agents to expand treatment options for specific pathogens in the future [148]. While current guidelines for antibiotic treatment duration are primarily based on expert consensus, further clinical trials are necessary to optimize treatment efficacy and minimize unnecessary drug exposure [149].

### Supportive therapy

Supportive care forms the foundation of treatment for purulent meningitis. This includes measures like temperature control, ensuring adequate rest, and providing necessary nutritional support. For children experiencing symptoms of increased intracranial pressure, interventions to reduce it become crucial. Similarly, antiepileptic therapy is necessary for those experiencing convulsions. Electrolyte imbalances or blood sugar abnormalities require close attention from the doctor, with adjustments to the treatment plan made promptly as needed. Adjunctive therapies, such as paracetamol, intravenous immunoglobulin, pentoxifylline, and a mixture of succinate, inosine, nicotinamide, and ribonucleotide, have been employed for certain children, although their efficacy remains uncertain [150]. In cases of *N. meningitidis* infection, isolation of the child during treatment is crucial to prevent transmission. This isolation serves as an important public health measure to control the spread of the disease and protect vulnerable populations.

### Immunotherapy

Glucocorticoids are not routinely recommended for the treatment of purulent meningitis in children; however, their reasonable application may mitigate inflammation and prevent adhesions. The results of a meta-analysis have shown that Children treated with corticosteroids for purulent meningitis had a significantly reduced risk of hearing loss (OR = 0.68, 95%CI 0.53–0.89, *P* = 0.004) and severe neurological sequelae (OR = 0.59, 95%CI 0.37–0.95, *P* = 0.03) [151]. This view has also been indicated by the European Society for Clinical Microbiology and Infectious Diseases (ESCMID), however, the American Academy of Pediatrics does not recommend glucocorticoid use in neonatal patients or as a routine treatment for children with pneumococcal meningitis but they are not recommended for use in neonatal patients or in routine use for children with pneumococcal meningitis by the American Academy of Pediatrics [152, 153]. Additionally, in cases of refractory purulent meningitis in children, the combined use of antibiotics and glucocorticoids has also been shown to have certain benefits [154, 155].

Preventive vaccination plays a critical role in controlling and preventing purulent meningitis. Vaccines targeting *N. meningitidis*, *S. pneumoniae*, *H. influenzae*, and other pathogens are widely used in clinical practice and have demonstrated significant effectiveness. For *N. meningitidi*s, various effective vaccines have been developed, including monovalent conjugate vaccines for serogroups A and C, as well as the quadrivalent meningococcal conjugate vaccine (MenACWY) for serogroups A, C, W, and Y, which have achieved significant success in multiple regions. In particular, the MenACWY vaccine is now recommended for broader protection [37, 156]. Additionally, the 4-component meningococcal serogroup B vaccine, which targets serogroup B and provides cross-protection against non-B serogroups, is suitable for all age groups and has been registered for use in over 50 countries worldwide [157]. Furthermore, novel vaccines under development, such as Meningococcal ACWYX conjugate vaccine and combined meningococcal ACWY and B conjugate vaccine, hold promise for better controlling *N. meningitidis* infections and transmission in the future [156]. For *S. pneumoniae*, primary prevention strategies include pneumococcal polysaccharide vaccines and conjugate vaccines. Among these, the 13-valent pneumococcal conjugate vaccine (PCV13), which covers multiple serotypes, is widely implemented in various countries and has shown good efficacy [158–161]. Studies emphasize the importance of rapidly expanding the use of PCV13 in low- and middle-income countries to achieve high coverage and maximize vaccine impact [162]. Meanwhile, the development of 20-valent pneumococcal conjugate vaccine provides additional protective options for children and has demonstrated good benefits in several countries [163–166]. However, while vaccine efficacy is crucial, potential adverse reactions must be rigorously monitored to ensure vaccination safety, and the impact of vaccine use on bacterial serotype epidemiology and antibiotic resistance patterns warrants careful consideration [141, 167, 168]. For *H. influenzae*, the Hib conjugate vaccine has been incorporated into immunization programs in most countries and has demonstrated significant efficacy [28, 169]. However, with the widespread implementation of the Hib vaccine, the incidence of non-Hib serotypes has increased, particularly *H. influenzae* serotypes a, e, and f [28, 170]. Therefore, continuous surveillance and updates to vaccination strategies are needed to adapt to evolving pathogen epidemiology and ensure effective prevention and management of related infectious diseases.

### Potential treatment

In recent years, researchers have been actively exploring strategies to improve drug delivery to the brain and overcome the BBB, a major obstacle in treating purulent meningitis. A recent study showed that *E. coli* can produce magnetic OMVs in the presence of magnetic iron oxide nanoparticles, which were able to improve survival and clinical behavioral scores in a mouse model of *E. coli* meningitis [171]. This discovery holds promise for drug loading and magnetic targeting across the BBB, potentially opening new avenues for treating purulent meningitis. Combination therapy with antibiotics and TLR2/TLR13 agonists has shown potential effectiveness against *S. pneumoniae* meningitis in mice models [172]. This approach has the potential to become a valuable adjunct to the treatment of purulent meningitis in the future. In addition, researchers are utilizing high-throughput DNA sequencing methods to identify potential immunotherapeutic targets for pathogens in newborn mice, laying a strong foundation for the development of novel immunotherapies [173]. While significant hurdles remain before clinical application, this approach offers a promising new weapon to combat antibiotic resistance. The role of NETs in bacterial infections is a growing area of research. Although in its early stages, targeting NETs may represent a novel therapeutic direction for purulent meningitis [111]. As scientific research continues to advance, we can be optimistic that the future of purulent meningitis treatment will be marked by increased accuracy and efficacy.

### Others

Beyond the treatment strategies discussed previously, several promising new avenues are emerging in the field of purulent meningitis therapy. One such area of interest involves the study of *E. coli* meningitis virulence factor Invasin of brain endothelium A (IbeA). Since IbeA disrupts the BBB, the development of IbeA inhibitors holds promise as an effective treatment to protect barrier integrity and potentially alleviate meningitis symptoms [64]. Another exciting area of research explores the therapeutic potential of short-chain fatty acids (SCFAs) – naturally occurring substances found on mucous membranes. These SCFAs, such as acetate, propionate, and butyrate, have been shown to inhibit pathogens. By regulating gut microbiota balance and enhancing mucosal immunity, SCFAs may offer novel treatment options by targeting the causative agents of purulent meningitis [174]. Similarly, natural compounds like andrographolide have demonstrated effectiveness against *K. pneumoniae* infections. These compounds may act through various mechanisms, including interfering with pathogen metabolism, inhibiting growth, or destroying cell structures, thereby providing new avenues for treating purulent meningitis [175]. Within the domain of personalized medicine, the columbaric model introduced by Liang et al. warrants particular attention. This model incorporates parameters like body weight, hemoglobin, red blood cell volume, WBC, monocyte count, prematurity, and a specific inflammation measure (logarithmic systemic immune-inflammation index) to provide clinicians with more accurate treatment recommendations. By predicting treatment response and optimizing regimens based on individual patient characteristics, this model has the potential to improve cure rates and quality of life for patients with purulent meningitis [176]. In conclusion, the field of purulent meningitis treatment is constantly evolving, with new approaches and strategies emerging. These ongoing studies equip us with a growing arsenal of tools to combat this serious neurological infectious disease.

## Outcome

Clinicians treating purulent meningitis must be vigilant in identifying risk factors that may indicate a poor prognosis. These factors can be analyzed from a patient’s clinical presentation, laboratory tests, and imaging findings. Clinical symptoms associated with poor prognosis include being younger than one year old, having dilated pupils in both eyes, a positive Babinski sign, altered consciousness, and a Glasgow Coma Scale score of less than 13. These symptoms reflect the severity of illness and nervous system involvement. Laboratory tests and imaging findings also provide valuable prognostic information. High levels of CRP in peripheral blood (≥ 50 mg/L), elevated WBC in CSF (> 500 × 10^6^/L), increased protein concentration in CSF (> 1.0 g/L), low CSF glucose content (< 1.5mmol/L), an elevated lactate dehydrogenase-to-albumin ratio (LAR), and an initial PCT level exceeding 0.1ng/dL upon admission are all potential indicators of poor prognosis. Additionally, hemoglobin levels below 90 g/L, abnormal head imaging findings, and abnormal EEG results during hospitalization may also suggest a poor outcome [177–181]. Beyond these factors, early ineffective antibiotic treatment, systemic complications, and alterations in the inflammatory response can further compromise a patient’s prognosis [182]. These factors highlight the importance of timely and effective treatment and the impact of a patient’s overall health on the disease course. In terms of risk assessment, Zhang et al. successfully developed a model for early prediction of risk factors in *E. coli* meningitis using relevant evidence, providing a powerful tool for clinicians [183]. Despite advancements in treatment, purulent meningitis can still lead to long-term disabilities, including cognitive impairment, hearing loss, hydrocephalus, and neurodevelopmental abnormalities. Hearing impairment has been associated with levels of CD64 and PCT in CSF (although the link to antibiotics remains controversial). Female gender, CSF glucose levels below 2 mmol/L, periventricular leukomalacia, stipular leukodystrophy, and empyema are considered risk factors for hydrocephalus [8, 9, 184–186]. The risk of abnormal nervous system development is particularly concerning in newborns, especially those with more severe clinical and neurophysiological manifestations [187]. Additionally, meconium-stained amniotic fluid and young age are significantly associated with poor outcomes in neonates [85, 188]. In conclusion, during the treatment of purulent meningitis in children, clinicians should be attentive to various risk factors that may indicate a poor prognosis and take appropriate measures to minimize the risk of long-term disabilities. Further research is necessary to improve our understanding and management of this serious neurological infection.

## Conclusion

Purulent meningitis is a serious and common infectious disease of the nervous system in children that can cause a variety of sequelae. Many different pathogens can cause purulent meningitis. Some of these pathogens spread through respiratory droplets, leading to a rapid onset of illness with little variation in timing across individuals. However, the specific types of pathogens may vary depending on geographic location. These pathogens can cross the BBB, damaging blood vessels in the brain and suppressing the body’s immune system. This can lead to hormonal imbalances and inflammation. Early diagnosis of purulent meningitis can be challenging because the initial symptoms are often non-specific. Currently, diagnosis of purulent meningitis relies primarily on a doctor’s experience (empirical judgment), laboratory tests, and imaging studies. Culturing bacteria from CSF remains the definitive diagnostic test (gold standard). Treatment for purulent meningitis typically involves a combination of general supportive measures, antibiotic therapy to target the specific pathogen, and sometimes immunotherapy. Vaccination has significantly reduced the incidence of purulent meningitis, representing a breakthrough in preventive medicine. Emerging therapeutic approaches, such as drugs that target bacterial virulence factors, hold promise but require further research and clinical trials for validation. In conclusion, significant challenges remain in diagnosing and treating purulent meningitis in children. Through ongoing research efforts focused on optimizing diagnostic and treatment methods, as well as improving prognosis assessment, we can strive to provide more accurate and effective care for children with this serious illness.

## Data Availability

Not applicable.

## Abbreviations

BBB: Blood-brain barrier
CRP: C-reactive protein
CSF: Cerebrospinal Fluid
CT: Computed tomography
EEG: Electroencephalogram
ESBLs: Extended-spectrum β-lactamases
ESCMID: The European Society for Clinical Microbiology and Infectious Diseases
*E. coli*: *Escherichia coli*
FIRST+: The Febrile Infant Triage Risk Score+
GBD: The Global Burden of Disease
GBS: Group B Streptococcus
Gli2: Glioma-associated oncogene homolog 2
HBP: Heparin-binding protein
Hib: Haemophilus influenzae serotype B
HH: Hedgehog
*H. influenzae*: *Haemophilus influenzae*
IbeA: Invasin of brain endothelium A
IDSA: Infectious Diseases Society of America
*K. pneumoniae*: *Klebsiella pneumoniae*
*L. monocytogenes*: *Listeria monocytogenes*
MenACWY: Quadrivalent meningococcal conjugate vaccine
mNGS: Next-generation metagenomic sequencing
MRI: Magnetic resonance imaging
NADPH: Nicotinamide adenine dinucleotide phosphate
NETs: Neutrophil extracellular traps
*N. meningitidis*: *Neisseria meningitidis*
OMVs: Outer membrane vesicles
PCR: Polymerase Chain Reaction
PCT: Procalcitonin
PCV13: 13-valent pneumococcal conjugate vaccine
RC: Rochester Criteria
SARS-CoV-2: Acute respiratory syndrome coronavirus 2
SCFAs: Short-chain fatty acids
SIRS: Systemic inflammatory response syndrome
*S. aureus*: *Staphylococcus aureus*
*S. pneumoniae*: *Streptococcus pneumoniae*
TGFBRII: Transforming growth factor beta receptor II
TLRs: Toll-like receptors
TNF-α: Tumor necrosis factor-α
WBC: White blood cell count
YOS: Yale Observation Scale
